# Retraction Note: Production of viable chicken by allogeneic transplantation of primordial germ cells induced from somatic cells

**DOI:** 10.1038/s41467-023-39170-5

**Published:** 2023-06-19

**Authors:** Ruifeng Zhao, Qisheng Zuo, Xia Yuan, Kai Jin, Jing Jin, Ying Ding, Chen Zhang, Tingting Li, Jingyi Jiang, Jiancheng Li, Ming Zhang, Xiang Shi, Hongyan Sun, Yani Zhang, Qi Xu, Guobin Chang, Zhenhua Zhao, Bing Li, Xinsheng Wu, Yang Zhang, Jiuzhou Song, Guohong Chen, Bichun Li

**Affiliations:** 1 Key Laboratory of Animal Breeding Reproduction and Molecular Design for Jiangsu Province, College of Animal Science and Technology, Yangzhou University, Yangzhou, China; 2 Joint International Research Laboratory of Agriculture and Agri-Product Safety of the Ministry of Education of China, Yangzhou University, Yangzhou, China; 3 The Poultry Research Institute of Chinese Academy of Agricultural Sciences, Yangzhou, China; 4 Department of Animal & Avian Sciences, University of Maryland, College Park, MD USA

**Keywords:** Biotechnology, Genetics, Molecular biology, Stem cells

Retraction to: *Nature Communications* 10.1038/s41467-021-23242-5 published online 20 May 2021

The authors have retracted this article. In this article, we reported that chicken embryo fibroblasts (CEFs) could be transdifferentiated to primordial germ cells (PGCs) and used to generate viable offspring after transplantation to chicken embryos. After publication, it was brought to our attention (by Marie-Cecile van de Lavoir and Philip Leighton of Omniab, Inc., Mike McGrew and Helen Sang of the Roslin Institute, and Benjamin Schusser of Technical University of Munich) that some of the analyses to support our conclusions regarding the origin of offspring were insufficient. Specifically, self-crossing, microsatellite and feather color data were thought to be inconclusive evidence that the offspring were donor derived. No concerns were raised regarding data integrity. However, additional review by two external experts concurred with the concerns raised, and we acknowledge that alterations to the experimental design would have allowed the origin of offspring to be determined with less ambiguity. The authors are performing further experiments and intend to submit a new manuscript for peer review in due course. All authors agree to this retraction.

